# A comparison between Asians and Caucasians in the dimensions of the femoral isthmus based on a 3D-CT analysis of 1189 adult femurs

**DOI:** 10.1007/s00068-021-01740-x

**Published:** 2021-07-28

**Authors:** Darius M. Thiesen, Dimitris Ntalos, Alexander Korthaus, Andreas Petersik, Karl-Heinz Frosch, Maximilian J. Hartel

**Affiliations:** 1 Department of Trauma and Orthopaedic Surgery, University Medical Center Hamburg-Eppendorf, Martinistr. 52, 20246 Hamburg, Germany; 2 Stryker Trauma GmbH, Kiel-Schönkirchen, Germany; 3 Department of Trauma, Orthopaedic Surgery and Sports Traumatology, BG Hospital, Hamburg, Germany

**Keywords:** Proximal femur fractures, Intramedullary nailing, Intramedullary implants, Anatomy femur, Femoral isthmus

## Abstract

**Introduction:**

For successful intramedullary implant placement at the femur, such as nailing in unstable proximal femur fractures, the use of an implant that at least reaches or exceeds the femoral isthmus and yields sufficient thickness is recommended. A number of complications after intramedullary femoral nailing have been reported, particularly in Asians. To understand the anatomical features of the proximal femur and their ethnic differences, we aimed to accurately calculate the femoral isthmus dimensions and proximal distance of Asians and Caucasians.

**Methods:**

In total, 1189 Asian and Caucasian segmented 3D CT data sets of femurs were analyzed. The individual femoral isthmus diameter was precisely computed to investigate whether gender, femur length, age, ethnicity or body mass index have an influence on isthmus diameters.

**Results:**

The mean isthmus diameter of all femurs was 10.71 ± 2.2 mm. A significantly larger diameter was found in Asians when compared to Caucasians (*p* < 0.001). Age was a strong predictor of the isthmus diameter variability in females (*p* < 0.001, adjusted *r*^2^ = 0.299). With every year of life, the isthmus showed a widening of 0.08 mm in women. A Matched Pair Analysis of 150 female femurs showed a significant difference between isthmus diameter in Asian and Caucasian femurs (*p* = 0.05). In 50% of the cases the isthmus was found in a range of 2.4 cm between 16.9 and 19.3 cm distal to the tip of the greater trochanter. The female Asian femur differs from Caucasians as it is wider at the isthmus.

**Conclusions:**

In absolute values, the proximal isthmus distance did not show much variation but is more proximal in Asians. The detailed data presented may be helpful in the development of future implant designs. The length and thickness of future standard implants may be considered based on the findings.

## Introduction

For optimized intramedullary implant placement in fracture care as well as in orthopaedic indications at the femur, the understanding of the exact anatomical characteristics is paramount. Next to the femoral torsion and antecurvation, the localization and dimension of the femoral isthmus is another detail that is important for the anchorage and stabilization of intramedullary implants [1–3]. In unstable fracture patterns at the proximal femur, for example, it is advisable to use an intramedullary implant that at least reaches or surpasses the femoral isthmus for optimized stability [4].

The femur was studied extensively in a multitude of publications, already. However, along with the evolution of technical possibilities, each scientific investigation was justified at its time of publication. As early as in 1914, Parsons described in his paper “The Characters of the English Thigh-Bone” the femoral anatomy using analogue measurements and drawings [5]. With the introduction of radiographic imaging, the femur was first studied using plain radiography and later on with computed tomography and digital photography [6–8]. Today, the technical progress allows 3D imaging and automated measurements to be used. With such novel technology, the femur itself has been studied already to investigate its accurate caput-collum angle [2], anterior curvature [9] and three-dimensional modelling of the femoral canal and simulation of the right nail entry point [10]. However, the femoral isthmus especially in an Asian population has only been studied once before with a 3D analysis in 204 Chinese patients. [3]. We present a study on 1189 computed tomography (CT) datasets originating from Asians and Caucasians femurs. The purpose of this investigation is the exact definition of anatomical properties of the femoral isthmus in this large cohort. Moreover, a subgroup analysis on possible gender and ethnic differences is presented. We hypothesize that there are different femoral isthmus dimensions between Asians and Caucasians.

## Methods

### Study population

A total of 1232 segmented 3D CT datasets of femur bones were analyzed. The data were collected by Stryker Trauma GmbH between 2008 and 2017 with the prior written consent of the patient. Personal data such as name, date of birth or date of CT associated with the datasets were removed and not provided to the company. CT scans that included the skull were also excluded, as described in our previous work [11]. The available demographic data generally included age, ethnicity, BMI and gender. In some patients BMI, age or gender were not available due to individual hospital data protection policies. All CT scans were reviewed to ensure exclusion of femurs containing implants, exhibiting pathology, malformations (tumorous, post-osteomyelitis, post-traumatic) and fractures. Correlation analyses were carried out using a total of 1232 adult femoral CT datasets. There were 820 (67%) of Caucasian origin, 369 Asian (30%), 28 African (2%), 14 Middle Eastern (1%) and 1 with unknown origin. Information on the age of the patients at the time of examination was available in 1146 of the cases with a mean age of 64 ± 16 years (range 18–109).

### 3D Modeling and analytics of bone morphology

A total of 1232 femurs were analyzed using a validated analytical software tool called SOMA. All CT scans were segmented using standardized protocols and commercial software (Mimics, Materialise NV, Belgium). In the next step, SOMA software was used to compute this large 3D sample in an automated and reproducible fashion [1, 2, 12].

The inner cortical wall which is the relevant geometry for the measurement of the isthmus diameter was determined by the density threshold of 580 HU [13].

### Determination of the femoral length

**Fig. 1:**
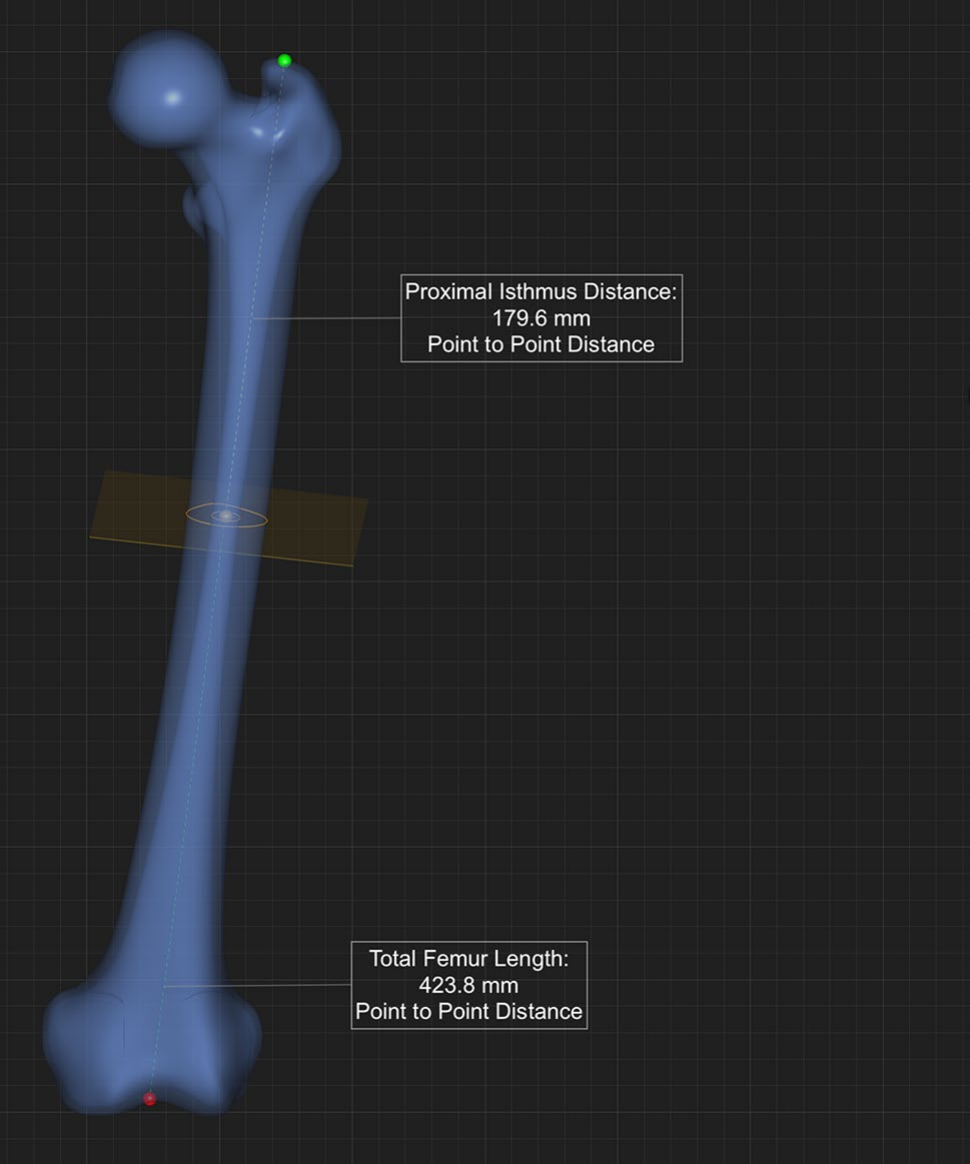
Shows the definition of the femoral length measured from tip of the greater trochanter to distal intercondylar notch

The length of the femur was determined as defined in previous work, measured from the tip of the greater trochanter to the distal intercondylar notch (saddle point of retropatellar joint) [1], also seen in Fig. 1**.**

### Localization of the femoral isthmus and determination its diameter

**Fig. 2:**
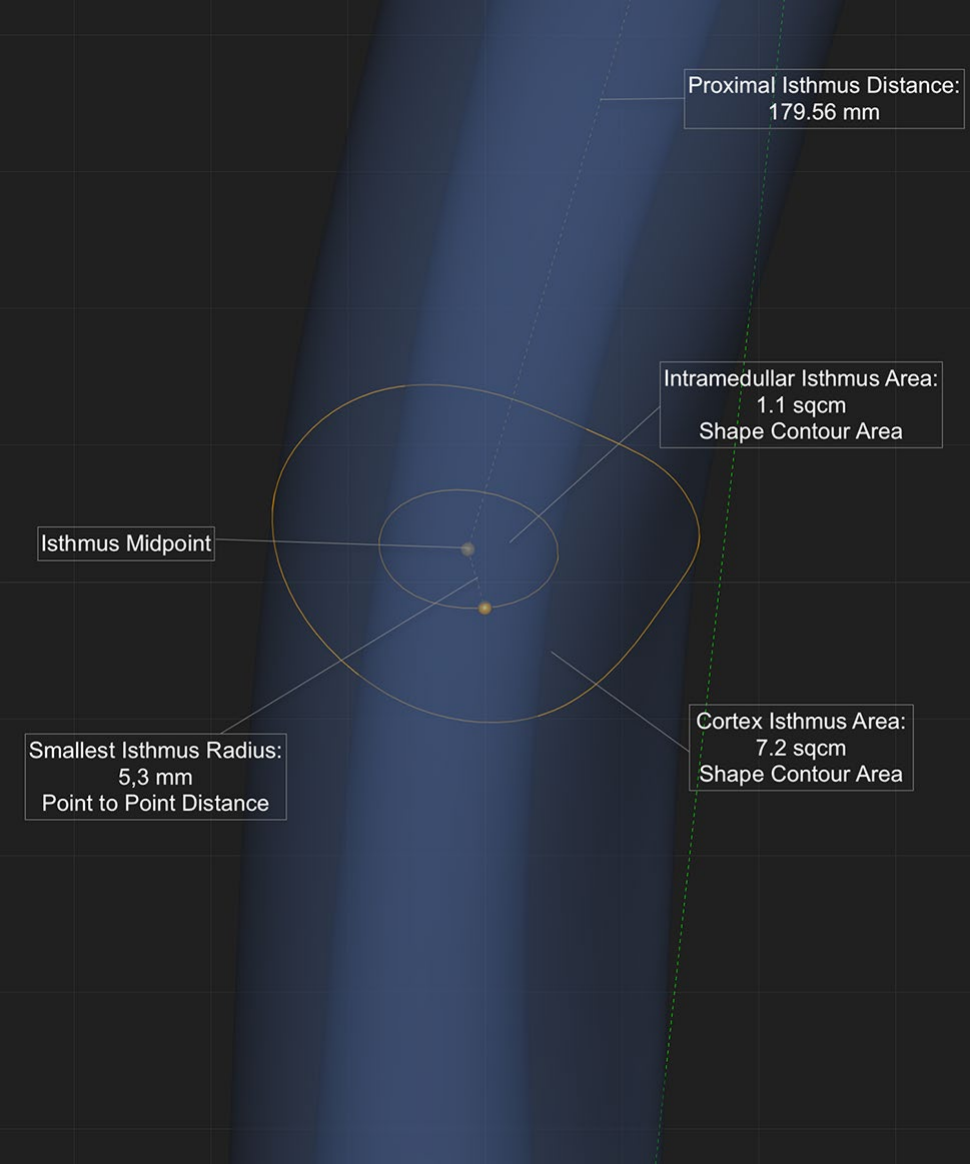
The orange dots mark the calculated proximal isthmus of the femur with the inner orange circle indicating the optimized inner cortical boundary. The distance of the two orange dots was defined as the isthmus radius, then multiplied by two for the isthmus diameter

In a first step, the center of the femoral canal was determined using inner cortical midpoints that connect to a line representing the curved anatomy of the femoral canal. The isthmus plane is perpendicular to the midline and located at the smallest radius found within the canal. The radius distance was multiplied by two to calculate the diameter of a best-fit circle at the isthmus plane. In a final step, the proximal isthmus distance was measured extending from a plane perpendicular to the femoral canal line at the level of the tip of the greater trochanter to the plane of the isthmus, see Fig. 2. The isthmus distance was measured from the exact midpoint of the femoral isthmus plane to the tip of the greater trochanter, defined before to measure the femoral length.

In addition, a calculation of the relative isthmus distance to the trochanter tip was performed, by dividing the absolute isthmus distance by the femoral length.

### Statistics

The continuous parameters are displayed as means with their standard deviations. With the Kolgorov-Smirnov-Test, the normal distribution of the data was calculated. For non-normal distributed data, non-parametric tests (Wilcoxon, Mann–Whitney-U) were used.

To calculate correlations between metric variables, Spearman’s rho was employed. Multiple linear regression analyses were performed to show the linear correlation of metric variables and their relative predictive impact.

After isolating the most powerful predictor of the isthmus diameter by performing a linear regression analysis, a Matched Pair analysis of female femurs of Asian and Caucasian individuals with the same age (± 2 years) was performed. A linear regression analysis was also performed in the same manner after dividing the subjects in male and female.

The level of significance for all statistical tests was 95% (alpha = 0.05). The Statistical analyses were all carried out using IBM SPSS V21 (IBM Corp., NY, USA). All methods were conducted in accordance with relevant guidelines and regulations. This study has been approved by our local ethics committee of (Ärztekammer Hamburg), WF -008/18.

## Results

### Descriptive statistics

There were 48% (*n* = 596) female cases. BMI was calculated using data on height and weight of 926 subjects. The mean BMI amounted to 24.9 ± 5 m^2^/kg with a range of 13.3–54. Please see Table 1 for more details on age, gender, ethnicity, BMI as well as height with relation to ethnicity. There were no differences in age and gender distribution between Asians and Caucasians, but Caucasians had a significantly higher BMI and they were taller (*p* = 0.001).

**Table 1:** Distribution of age, gender, ethnicity, BMI as well as height with relation to ethnicity

Ethnicity	Gender *n* = 1189	Age, mean ± SD, (range), years *n* = 1146	BMI mean ± SD, (range), m^2^/kg *n* = 926	Height mean ± SD, (range), cm *n* = 926
Asian	Male = 177 Female = 192 *n* = 369	59.1 ± 17.9, (18–91) 69.2 ± 18.7, (19–96) 64.5 ± 18.9	24.1 ± 3.3 (16–34) 22.2 ± 4.1 (13–39) 22.9 ± 3.9	169.8 ± 7 (149–182) 152.6 ± 7.5 (130–169) 159 ± 11.1
Caucasian	Male = 433 Female = 385 *n* = 818	63.7 ± 14.3, (19–109) 65 ± 15.5, (19–96) 64.3 ± 14.9	26.2 ± 4.9 (14–53) 26.5 ± 6.2 (14–55) 26.3 ± 5.6	175.1 ± 7.6 (154–199) 164.8 ± 7.6 (142–192) 170 ± 9.2
*p* value *Caucasian and Asian*	0.61	0.2	0.001	0.001

### Isthmus diameter and distance

The mean isthmus diameter of all femurs was 10.71 ± 2.2 mm. A significantly (*p* < 0.001) larger diameter was found in the Asian population when compared to Caucasians as depicted in Table 2. The mean female Caucasian isthmus diameter was 10.1 ± 2.3 mm. It was found to be significantly smaller when compared with both, the mean diameter of male and female Asian as well as male Caucasians (*p* < 0.001). When comparing male femurs only, no significant differences between Asians and Caucasians regarding the isthmus diameter were found (*p* = 0.598).

**Table 2:** Isthmus diameters, proximal isthmus distances and ratio of isthmus distances to femur length divided by gender and ethnicity

	Isthmus Diameter., mean +—SD, (range), mm	Median (IQR), mm	Isthmus Distance, mean +—SD, (range), mm	*Median (IQR)*	Femur Length, mean +—SD, (range) mm	*Median (IQR)*	Ratio Isthmus Distance to Femur Length, mean +—SD, (range)	
All Femurs, *n* = 1232	10.71 ± 2.2 (5.6–19.4)	10.6 (9.2–12)	181 ± 22 (100–276)	180 (169–193)	420 ± 31 (329–508)	421 (398–442)	43 ± 6 (25 61)	42 (40–46)
Female, *n* = 594	10.4 ± 2.3 (5.6–18.4)	10.2 (8.6–11.8)	174 ± 21 (100–242)	173 (161–185)	402 ± 26 (329–502)	401 (384–420)	43 ± 5 (25 61)	43 (20–46)
Male, *n* = 636	11 ± 2.1 (5.8–19.4	10.8 (9.4–12	188 ± 21 (124–276)	185 (176–200)	437 ± 25 (363–508)	437 (419–454)	43 ± 4 (28 61)	42 (40–45)
*p-value*	< *0.001*		< *0.001*		< *0.001*		0.09	
Asian, *n* = 369	11.1 ± 2.1 (6–18.8)	10.8 (9.6–12.2)	173 ± 23 (100–257)	174 (160–186)	398 ± 27 (329–467)	397 (379–418)	43 ± 5 (25 61)	43 (20–47)
Caucasian, *n* = 818	10.6 ± 2.3 (5.6–19.4)	10.4 (9–12)	185 ± 21 (115–276	183 (172–197)	430 ± 28 (353–508)	430 (410–450)	43 ± 4 (27 61)	42 (40–45)
*p-value*	< *0.001*		< *0.001*		< *0.001*		*0.004*	
Asian Females, n = 193	11 ± 2.2 (6–17.8)	10.8 (9.4–13)	167 ± 22 (100–217)	167 (155–181)	380 ± 18 (329 – 435)	381 (367 – 392)	44 ± 6 (25 – 60)	44 (41–48)
Caucasian Females, *n* = 384	10.1 ± 2.2 (5.6–18-4)	9.8 (8.4–11.6)	177 ± 19 (115–242)	175 (166–189)	413 ± 22 (352 – 402)	413 (398 – 428)	43 ± 4 (27 61)	42 (40–45)
*p-value*	< *0.001*		< *0.001*		< *0.001*		*0.002*	
Asian Males, *n* = 177	11.08 ± 1.9 (7–18.8)	11 (9.6–12)	181 ± 21.3 (124–257)	178 (170–194)	418 ± 20 (363 – 467)	418 (404 – 431)	43 ± 5 (27 61)	43 (40–46)
Caucasian Males, *n* = 433	10.98 ± 2.1 (5.8–19.4)	10.8 (9.4–12.4)	192 ± 19.5 (127–275)	188 (179–202)	446 ± 22 (371 – 508)	446 (430 – 460)	43 ± 4 (28 56)	42 (40–45)
*p-value*	0.598		< *0.001*		< *0.001*		*0.392*	

Statistically significant *p* values are in italics (*p* < 0.05)

In the gender subgroup analysis, a significantly larger diameter was observed in male when compared to female subjects, *p* < 0.001 (Table 2).

**Fig. 3:**
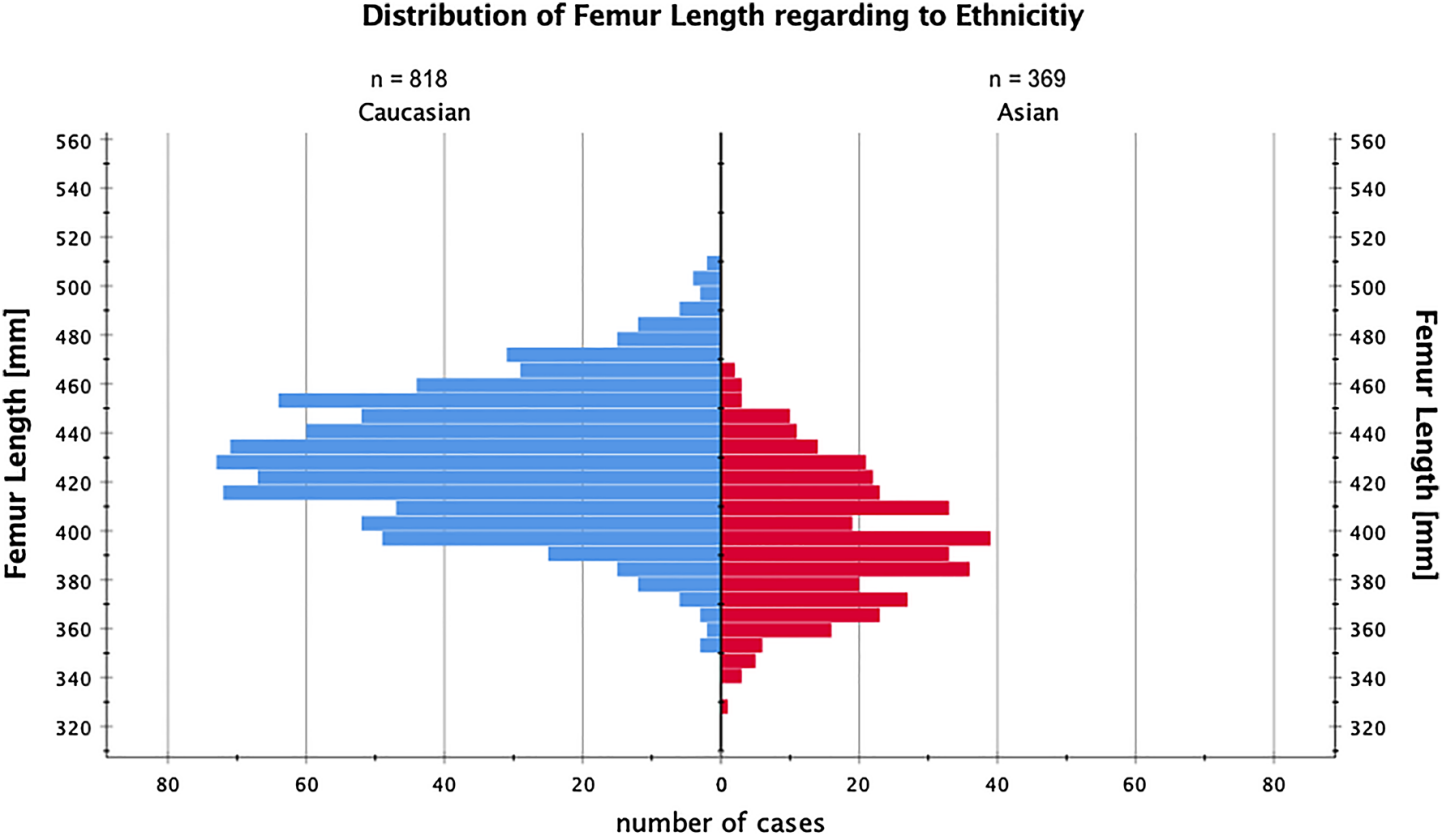
Shows the distribution of femur length and its distribution in our cohort. On the y-axis, the femur length is shown in millimeters and on the x-axis, the absolute number of cases is shown as red (Asian) or blue (Caucasian) bars

**Fig. 4:**
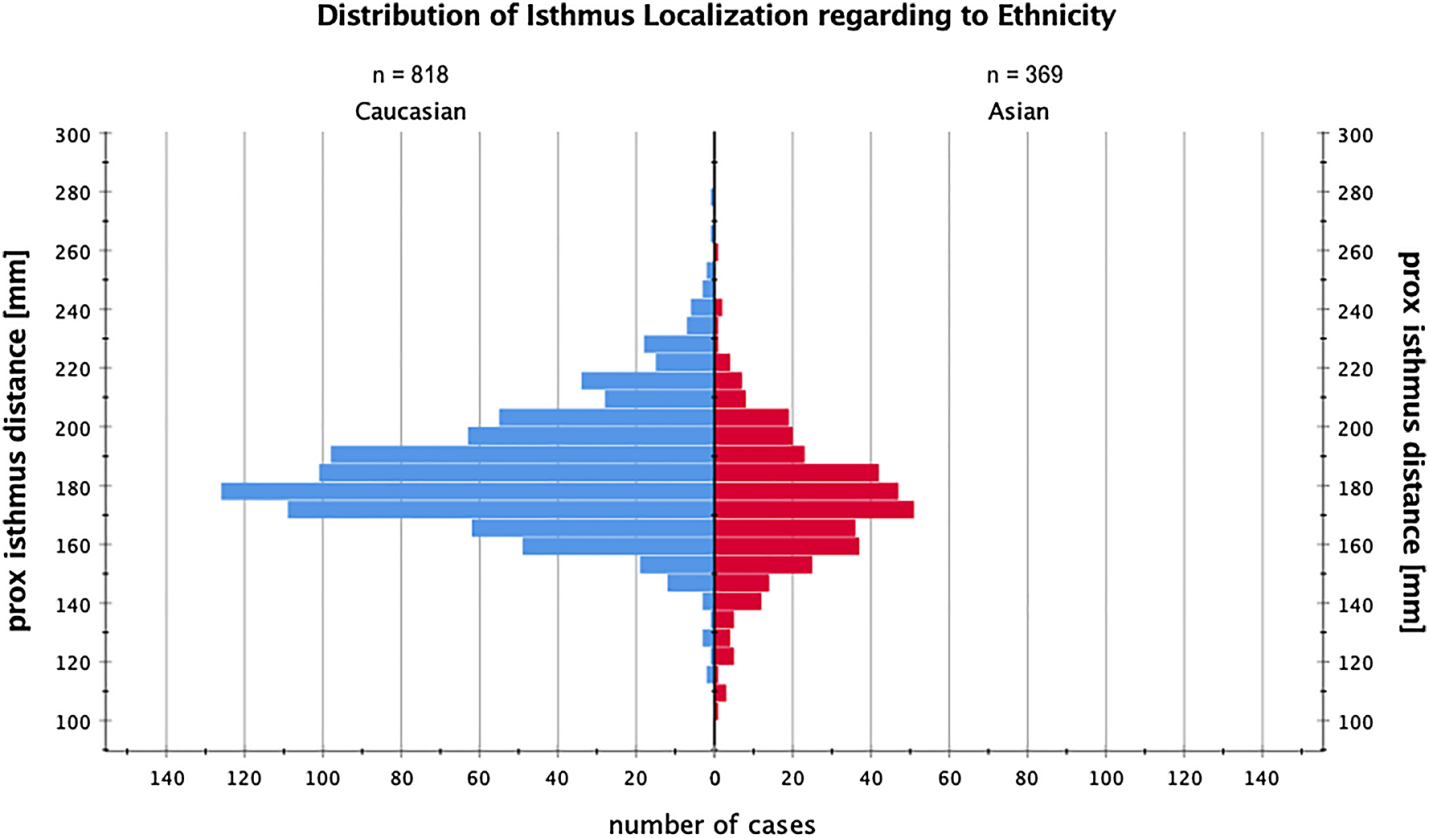
Shows the distribution of the isthmus distance and its distribution in our cohort. On the y-axis, the isthmus distance to the greater trochanter tip is shown in millimeters and on the x-axis, the absolute number of cases is shown as red (Asian) or blue (Caucasian) bars

Between men and women, Asians and Caucasians, female and male Caucasians to female and male Asians there was a significant difference in absolute isthmus distance (*p* < 0.001), which is shown in Table 2. The mean value of the isthmus distance (isthmus distance = ID) was 181 ± 22 mm for the whole population. The interquartile range of the ID was 169–193 mm, which means that in 50% of the cases the isthmus was found in a range of 2.4 cm between 16.9 and 19.3 cm proximal to the tip of the greater trochanter. In 90% of the femurs, the isthmus was found between 14.8 and 23.8 cm proximal to the tip of the greater trochanter, see also Figs. 3 and 4 for a visualization of the distribution of the ID and femur length between the ethnicities. The shortest quartile of femurs (328–398 mm) yielded a mean femur length of 380 ± 14 mm, and a mean ID of 169 ± 19 mm (100–240), while the longest quartile of femurs (442–508 mm) yielded a mean length of 460 ± 14 mm and a mean ID of 195 ± 21 mm (127–275 mm). The isthmus distance was significantly different between these two quartiles (*p* < 0.001).

### Correlation analysis of isthmus diameter and isthmus distance

These analyses were performed exclusively with Caucasian and Asian femurs (*n* = 1,187). The Isthmus diameter correlated moderately with age and proximal isthmus distance, measured from the greater trochanter (Spearman’s rho, *r* = 0.37 and 0.3, respectively; *p* < 0.01).

Between femur length, BMI, gender and cortical area a weak correlation was found (Spearman’s rho *r* = 0.2, *p* < 0.01).

A stepwise linear regression analysis demonstrated that age, gender, height, weight, BMI and proximal isthmus distance to trochanter tip explain 23.2% of the variability of the isthmus diameter (*p* < 0.001, adjusted *r*^2^ = 0.232) with no or a weak effect. All other variables had no significant impact on the isthmus diameter. The unstandardized beta for age was 0.025 (CI 0.2–0.29). This means that with every year of age the isthmus diameter will grow by 0.025 mm. When dividing the participants by gender the linear regression analysis for females showed that age alone explained 29% of the isthmus variability (*p* < 0.001, adjusted *r*^2^ = 0.299) and was a weak predictor. In females, the unstandardized beta for age was 0.08 (CI 0.2–0.29) leading to a growth of 0.08 mm of the isthmus by every year of life.

Finally, a Matched Pair Analysis of 150 female femurs showed a significant difference between isthmus diameter in Asian and Caucasian femurs, *p* = 0.05.

The Pearson correlation showed that the proximal isthmus distance correlated weakly (*p* < 0.001) with body height (*r* = 0.345) and femur length (*r* = 0.467). Regression analysis showed that ethnicity, gender, BMI, height and weight did not influence the isthmus distance ratio (IDR). Age and isthmus diameter had no influence (*r*^2^ = 0.11, *p* < 0.001) on IDR.

## Discussion

In this study, a thorough analysis of the localization and dimensions of the femoral isthmus is presented. Two different ethnic groups are compared. The most important finding in this study are small but significantly different isthmus diameters and positions between Asian and Caucasian femurs. Particularly Caucasian female femurs are thinner at the femoral isthmus, when compared with Asians. It was also shown that the isthmus localization in Asians is usually found 12 mm more proximally. To achieve optimized inner cortical contact surface between implant and bone for maximized stability, the information on isthmus localization and diameters may be helpful to both clinicians and implant developers.

It was described before, that the Asian femoral anatomy is different from Caucasians [10, 14–17]. Interestingly, in our cohort the femur length did not have the most significant influence on the diameter. Asians had wider isthmus diameters although they were smaller and had shorter femurs. When looking at studies examining the proximal Asian femur, there is a variation of methodologies and results. For example, Xiu-Yun and others [3] describe in their study with 204 healthy Chinese patients an average isthmus diameter of 10.05 ± 1.71 mm in 63 women of unknown age and height, which is lower than in our cohort. Although they used a precise 3D technology similar to our methodology, detailed information about their population is missing. In contrast to our and other authors [16, 18, 19] findings, they found no influence of age on the isthmus diameter. Though, femur length correlated weakly.

With the aim of developing new stem designs for the Asian population, there are several studies of proximal femoral anatomy [17, 20–23], that could contribute to the understanding of the diverse femoral shape. Some authors [14, 16] described a higher incidence of “champagne flute”-like proximal femoral canals in Malaysian and Chinese populations as defined by the Canal Flare Index (CFI) of more than 4.7 according to Noble and Dorr [24, 25]. Pi and colleagues [16] measured 500 radiographs of Chinese femurs before total hip arthroplasty and compared them with Western data, concluding that the “champagne flute” type is more common in Asians. Despite the large number of patients, comparison with our results is difficult because they used 2D radiographs and did not report their absolute values in English.

This indicates a wide metaphyseal region but a narrower canal 2 cm below the lesser trochanter. The higher incidence of Dorr type A proximal femurs (“champagne flutes”) in some Asian populations would indicate a narrower isthmus surrounded by thicker cortical walls.

However, in a study of Baharrudin and colleagues [14], 3D CT measurements were performed in a healthy and very young population with a mean age of 25 ± 5 years of only 30 female participants, which makes comparison challenging. The authors measured via conventional CT scans with a slice thickness of 3 mm and a low resolution but created an exact 3D model of the medullary canal. They found the isthmus to be 9.7 ± 1.7 mm at its smallest diameter which seems plausible as their population was quite young.

It could be possible that cortical degradation during the ageing process takes place to a greater extent in Asians than in Caucasians. However, there are no valid longitudinal studies in the literature to support this yet.

Femoral isthmus widening during the ageing process was described by several authors for Caucasian [19, 26] (X-ray) and for Japanese or Chinese [16, 27, 28] (X-ray, 3D CT, 3D CT) populations with different measurement methods. It appears that the effect of cortical deterioration at the isthmus level in women is more pronounced than in men. Age in particular seems to be the strongest predictor in both ethnic groups. Our data support the results of Milligan et al. [19]. The amount of 0.08 mm isthmus widening per year was exactly the same in female patients, although Milligan’s results were based on calibrated X-ray measurements and ours on 3D CT-data.

A higher incidence of postmenopausal osteoporosis is thought to explain the increased bone loss in women compared to men, which is also reflected in higher rates of hip fractures in women worldwide [29, 30]. However, as our matched pair-analysis suggests, there may be other factors apart from osteoporosis that contribute to alterations in isthmus diameters between different ethnic groups.

As mentioned in the introduction, the nature of the femur has been studied extensively already. However, unanswered questions may be resolved with the use of novel technologies. Zhao et al. for example, state in their study, that current nail designs have the potential to cause complications and further improvements are required for the use in Asians [31]. We were able to show that the relative proximal isthmus distance to the tip of the trochanter is more or less the same in both the Caucasian and Asian group. Nevertheless, due to the smaller body size and shorter femurs in Asians, the isthmus is located about 12 mm proximally, closer to the tip of the greater trochanter. In 50% of the Asians in our cohort, the isthmus was located in an area of 2.6 cm between 16 and 18.6 cm distal to the tip of the greater trochanter. When comparing the longest versus the shortest quartile of femurs the isthmus is located at 19.5 versus 16.9 cm distal to the tip of the greater trochanter even though the femoral length discrepancy is 8 cm. This is probably due to the more extensive length growth of about 70% in the distal part of the femur  [32].

Surgeons may find the information presented in the paper useful for unstable fracture cases where optimized intramedullar stability is desired in proximal femoral nailing: Depending on the individual patients CCD and the caphallomedulary angle of the implant of choice, the implant may stand out proximally which needs to be additionally taken into account when choosing the implants length [2, 33]. We found a mean overall isthmus distance of 181 ± 22 mm, measured from the proximal greater trochanteric tip. Practitioners can expect the isthmus to be further away in Caucasian men and nearer by in Asian women. According to the findings in this investigation, in a number of cases, a nail model with a length of 200 mm and more may be more suitable for optimized intramedullar fit.

We also did not find a significantly narrower canal in Asians which could cause additional problems. On the contrary, especially older Asian women had on average a wider isthmus. In summary, these findings indicate that currently available trochanteric standard nails will reach the isthmus in the majority of the cases, especially in Asian populations. However, the implant thickness required may differ between ethnic subgroups. Finally, in some of the cases a longer implant will be required to reach the isthmus for improved stability of the construct.

This study has the following limitations: The comparison of the isthmus diameter and its localisation between the Asian and Caucasian cohort was restricted by the slightly different age distribution in these groups. Therefore, an additional matched pair analysis was carried out. In this study, a general symmetricity is assumed for healthy and uninjured femurs (left vs right). In the literature, this has yet been shown to be true at the proximal femur [16]. Data on weight and height were not available in 306 patients and data on age in 86 cases, consequently these patients were excluded in the linear regression and the matched pair analysis.

As we have analysed a large data set of *n* > 1000 femurs, care must be taken in the interpretation of statistically significant differences. The differences found to be significant may not be clinically relevant in every case. In any case however, the improved precision of the data will likely be found useful in future implant generations.

The study presented in this paper is the first in the literature to report precise machine-measured differences in isthmus diameters between Asians and Caucasians from a very large dataset. The detailed data presented in this work will be helpful in the development of future implant designs. The length and thickness of future standard implants may be reconsidered based on the findings.
